# Evaluation of an objective staging system for assessment of cervical vertebral maturation

**DOI:** 10.1186/s12903-023-03844-9

**Published:** 2024-01-17

**Authors:** Ahlam M. Alhamady, Ramy Abdul Rahman Ishaq, Maged S. Alhammadi, Abeer A. Almashraqi, Najah Alhashimi

**Affiliations:** 1 Master of Science, Orthodontics, Faculty of Dentistry, University of Science and Technology, Sana’a Yemen, Sana’a, Republic of Yemen; 2 Department of Orthodontics, Pedodontics and Preventive Dentistry Faculty of Dentistry, Sana’a University, P. O. Box 271, Mathbah, Sana’a Yemen, Sana’a, Republic of Yemen; 3 Orthodontics and Dentofacial Orthopedics, Department of Preventive Dental Sciences, College of Dentistry, Jazan University, Jazan, Saudi Arabia; 4 Department of Clinical Oral Health Sciences, College of Dental Medicine, QU Health, Qatar University, Doha, Qatar; 5 Unit and Divisional Chief Orthodontics at Hamad Medical Corporation and Associate Professor at College of Dental Medicine, Qatar University, Doha, Qatar

**Keywords:** Cervical vertebral maturation, Objective staging, Superior wall inclination angle, Skeletal maturation, Orthodontics

## Abstract

**Background:**

The aim of this study was to evaluate an objective method for Cervical Vertebral Maturation (CVM) staging.

**Methods:**

An initial sample of 647 Lateral Cephalometric Radiographs (LCR) were staged according to the CVM (Baccetti et al.) by 4 examiners. The final sample (n = 394) included LCR on which the staging of the 4 investigators matched. The objective staging was performed by a single operator. The sample was divided according to the maturational stages into pre-pubertal, pubertal and post-pubertal groups. Measurements were performed on the cervical vertebrae (C2, C3 and C4). The angle between posterior and superior borders for C3 and C4 was the Superior Wall Inclination Angle (SWIA). Concavity Depth (CD) for C2, C3 and C4, and Body Shape (BS) (ratio of width to height of C3 and C4). Measurements of the 3 groups were compared.

**Results:**

Reliability of subjective staging was high (intra-observer reliability, 0.948; inter-observer reliability, 0.967). Good agreement was observed for the outcomes measured. Intra-observer reliability was good (0.918, 0.885 and 0.722 for CD, BS and SWIA, respectively). The same was for the inter-observer reliability results (0.902, 0.889 and 0.728 for CD, BS and SWIA, respectively). Significant differences were observed for mean values of SWIA and BS and median values of CD within maturational stage. Similar findings were observed when the outcomes were compared at different phases (*P* < 0.001).

**Conclusions:**

A standardized, objective staging system using linear, angular measurements and ratios was applied for the determination of cervical vertebral maturation.

## Background

Establishing a treatment plan for growing patients with craniofacial skeletal imbalances is modified by the growth status of the patient [1]. Detection of individually variable timings of Peak Height Velocity (PHV) of mandibular growth and the Pubertal Growth Spurt (PGS) require a reliable indicator that would provide accurate data including skeletal maturation and bone age to enhance the decision making process for orthodontic treatment planning, timing and prognosis of the used corrective therapy, functional appliance treatment [2]. 

The currently common approach for evaluation of skeletal maturation is the Cervical Vertebral Maturation (CVM) from Lateral Cephalometric Radiographs (LCRs), an essential diagnostic orthodontic record. This way, the patient is not exposed to additional radiographic radiation [3, 4]. CVM was pioneered by Lamparski [5] in the form of an atlas characteristic features. It was based on the initiation and development of lower border concavities and the increased development in height of the Cervical Vertebrae (CV) [6]. Hassel and Farman introduced modifications by limiting the assessment to the second, third and fourth CV [7]. Further modifications were introduced by Baccetti et al. by merging stages 1 and 2 (5 stages method) [8]. In 2005, an improved version of the CVM was proposed by Baccetti et al. to detect the peak mandibular growth from 6 stages (2 pre-pubertal, 2 pubertal and 2 post-pubertal) [9].

Maturation or cervical stages (CS) are differentiated by CV morphological features; inferior border of C2, C3 and C4 would be flat at the pre-pubertal stages and eventually, concavities start to develop and become more accentuated as an individual matures to the pubertal and post-pubertal stages. The shape of the CV body of C3 and C4 is another feature used to identify the stage of maturation. The shape would be trapezoidal with tapered superior border, then it would change with maturation to horizontally rectangular, squared and vertically rectangular shapes at the pubertal and post-pubertal stages, respectively [9].

The reproducibility of CVM is controversial [10, 11]. Some studies reported good reproducibility [12, 13], while others reported reproducibility issues [14, 15]. The subjective description of the stages resulted in disagreement between the observers [15]. This subjectivity was reported to cause low inter-rater agreement (less than 50%) [16]. The reported poor reproducibility was attributed to training level [17], clinical experience [18] and the assessment method itself [11].

Objective assessment of CVM was proposed with the aim of avoiding the subjectivity of the visual methods. Mito et al. developed a regression formula based on measurements on C3 and C4 [19]. Chen et al. established an equation for quantitative evaluation of CVM on 87 children and adolescents from 8 to 18 years old [20]. Beit et al. calculated skeletal age based on morphometric changes of the vertebral bodies C2 through C4, they concluded that the formula was not accurate enough for skeletal age estimation [21]. A previous study [22] assigned measurements to the morphological features of CV according to the method of Hassel and Farman [7] and was not validated. Furthermore, Cervical Vertebral Body’s Volume was introduced as a new parameter to evaluate CVM objectively from Cone Beam Computed Tomography (CBCT) images [23]. A recent study by Chandrasekar et al. derived formulae to objectively evaluate CVM in Asian South Indian male and female patients, the formulae derived were validated and found reliable for objective determination of CVM for both genders [24]. Machine learning or deep learning algorithms have been used for detecting CVM on LCRs [25, 26].

Most formulae postulated for determination of CVM were described as cumbersome. Manual application increased the chance for error [24]. In this regard, it was suggested to incorporate the formula(e) into computerized tracing programs to facilitate practical application [24, 27]. However, not all clinicians use digital tracing programs. In a questionnaire, the use of digital LCR was reported by 68.47% of the participating clinicians [28]. The application of artificial intelligence carried their limitations, and required additional hand-crafted features, which is time-consuming, and it heavily relied on the quality of initial manual landmark localization [3]. From the above, it may be stated that the documented subjectivity of subjective CVM methods and the complexity of objective methods provide rationale for further research.

The research question that prompted the study is: Are there measurable parameters in cervical vertebral morphology to objectively distinguish the stages of maturation and hence estimate the skeletal age of growing orthodontic patients? According to the null hypothesis, statistical differences between outcomes measured in the maturation stages would not be detected. The study aimed to answer the research question and test the proposed hypothesis by evaluation of an objective staging system for staging of CVM based on standardized measurements. Three outcomes were evaluated; the Superior Wall Inclination Angle (SWIA) was the primary outcome. Additional outcomes included the Concavity Depth (linear measurement) and Body Shape (ratio of width to height of CV body).

## Methods

### Study design and data source

This retrospective cross-sectional study was conducted in accordance with the European guidelines on radiation protection in dental radiology. The study was approved by the Ethics Committee of the College of Dentistry, University of Science and Technology, Sana’a, Republic of Yemen (ECA/UST198).

The sample was obtained from the database of a private radiology center (Al-Waleed Radiology Center, Sana’a, Republic of Yemen). Radiographs were selected according to specific initial inclusion criteria: (1) 8 to 19 years old; (2) Class I skeletal anteroposterior relationship (ANB = 2–4°) and average vertical proportions (MMPA = 26 ± 5°); (3) LCR taken using the same x-ray machine; (4) proper head orientation (Frankfurt horizontal plane was parallel to the true horizontal). Exclusion criteria included (1) radiographs with artifacts or failed to clearly show the area of interest (C2, C3 and C4); (2) LCRs with radiographically evident craniofacial anomalies; (3) history of orthodontic treatment or reported trauma or surgery to the head and neck region. Informed consent was obtained from all subjects and/or their legal guardian(s). They were contacted, it was explained that their radiographs were required for research purposes with no further obligation from their side and no need for further radiographs.

**Fig. 1:**
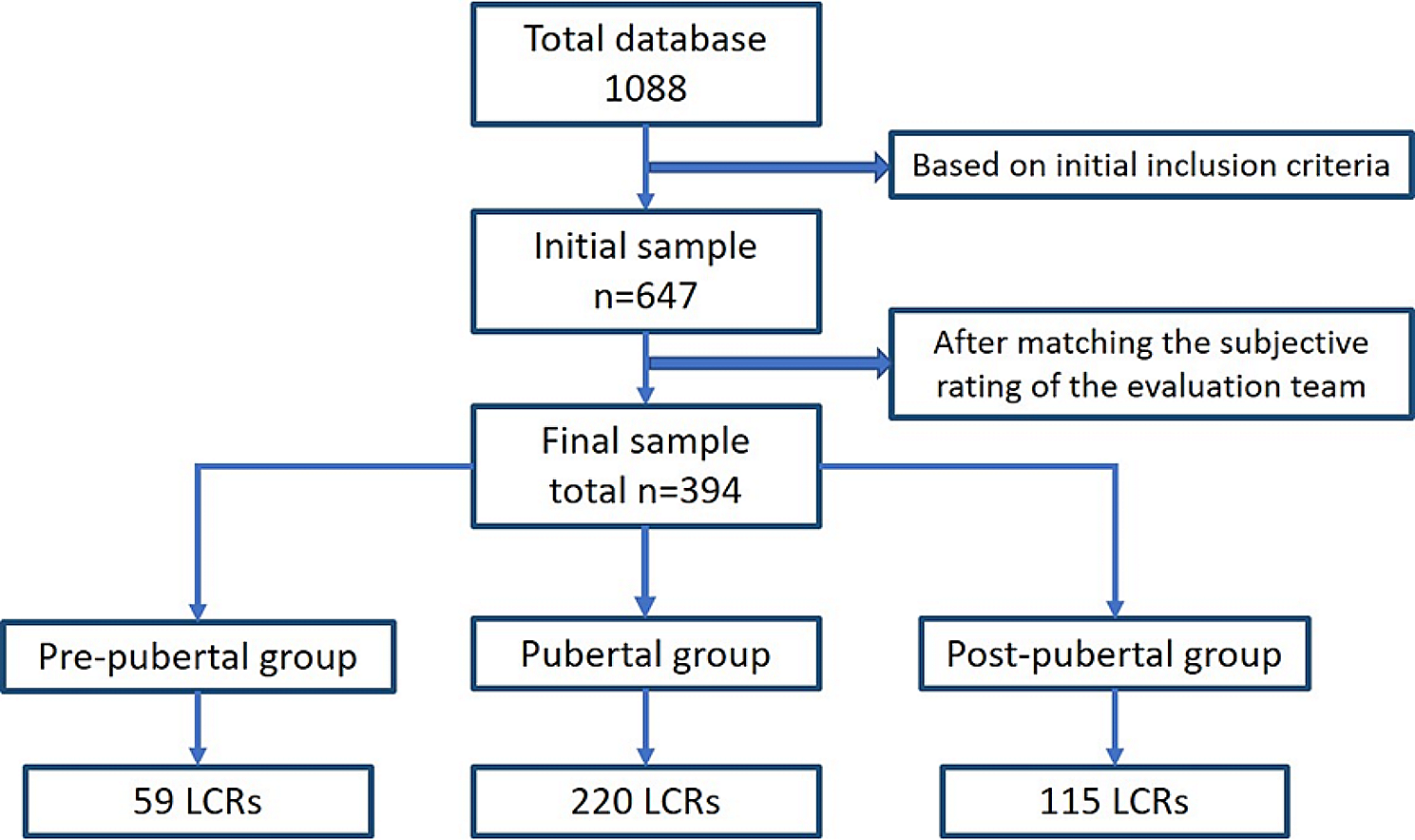
Flowchart demonstrating the flow and distribution of the selected sample

All the radiographs were obtained with the same cephalometric machine (PaX-Flex3D P2, Vatech, Korea) with a magnification factor of 1.4. Initially, radiographs were included based on a wide chronological age range. To ensure uniform distribution of the sample, 6 age groups (A-F) were created. Group A was assigned for radiographs obtained from 8 to 9 years old, group B was for 10–11 years old. Consecutive groups C-F included 2 years age groups. The distribution is presented in Fig. 1. From an initial database of 1088, 647 LCRs were included in the study.

### Subjective assessment

Subjective assessment was based on the method of Baccetti et al. [9]. The method described pre-pubertal (CS1 and CS2), pubertal (CS3 and CS4), and post-pubertal (CS5 and CS6) stages of maturation. Radiographs were prepared for evaluation by a blinded independent researcher. The images were coded and cropped to the area of the CV. The cropped images were randomly arranged in a PowerPoint slide show for presentation to the evaluation team. The team included four calibrated evaluators: the primary researcher (A.A), two experienced orthodontists (R.I and M.A) and an oral and maxillofacial radiologist (A.A.A). All members had more than 10 years of experience in the assessment of CVM. Initially, the staging system was revised in two training sessions. The rating was tested for intra and inter-observer reliability by evaluation of 10% of the sample twice within a two-week interval. Each evaluator was then required to independently assign a CVM stage to each image for the whole sample.

To be approved for inclusion, a LCR was required to score agreement on the designated CVM stage between the 4 evaluators. A Total 394 LCRs were prepared for the objective staging.

### Objective staging

The coded images were then prepared for the objective staging by an independent researcher in the form of paper prints (with a 1:1 magnification factor). The sample was divided into three groups according to the subjective assessment performed earlier by the evaluation team. LCRs of stages CS1 and CS2 were included in the pre-pubertal stage. The pubertal stage included LCRs from stages CS3 and CS4 and the post-pubertal stage included LCRs from stages CS5 and CS6.

**Fig. 2:**
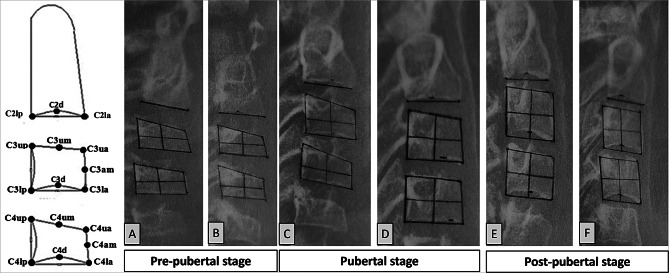
Image to the left is the diagrammatic representation of the objective staging system points; (**A**–**F**) Samples from the hand tracing and point identification of the original sample for the pre-pubertal stage (**A** and **B**), pubertal stage (**C** and **D**) and post-pubertal stage (**E** and **F**)

Tracing, point identification and measurements were conducted by an orthodontic resident (A.A). On each film, the second, third and fourth CV were hand traced using a 0.5 mm lead pencil and 8x10” matte acetate paper (0.003 mm thickness). On the tracing, 17 points were marked on C2, C3 and C4 (Table 1). Planes were then constructed from the points created as demonstrated in Fig. 2; Table 1.

**Table 1:** Desciption of the points and planes used in the objective staging system

No.	Point	Description
1	C3lp	The most posterior point on the inferior border of the body of C3.
2	C3ua	The most anterior point of the superior border of the body of C3.
3	C3up	The most posterior point of the superior border of the body of C3.
4	C4lp	The most posterior point on the inferior border of the body of C4.
5	C4ua	The most anterior point of the superior border of the body of C4.
6	C4up	The most posterior point of the superior border of the body of C4.
7	C2d	The most superior point of the inferior border of the body of C2.
8	C3d	The most superior point of the inferior border of the body of C3.
9	C4d	The most superior point of the inferior border of the body of C4.
10	C2la	The most anterior point on the inferior border of the body of C2.
11	C2lp	The most posterior point on the inferior border of the body of C2.
12	C3la	The most anterior point on the inferior border of the body of C3.
13	C4la	The most anterior point on the inferior border of the body of C4.
14	C3um	The middle point on the superior border of the body of C3.
15	C3am	The middle point on the anterior border of the body of C3.
16	C4um	The middle point on the superior border of the body of C4.
17	C4am	The middle point on the anterior border of the body of C4.”
	**Plane**	**Description**
1	CL2, CL3, CL4	Connecting points Cla to Clp at the lower border of each CV.
2	CS3, CS4	Superior border of C3 and C4 connecting points Cua and Cup for C3 and C4
3	CA3, CA4	Anterior border of C3 and C4 connecting points Cua-Cla for C3 and C4
4	CP3, CP4	Posterior border of C3 and C4 connecting points Cup-Clp for C3 and C4
5	BW	Width of CV body, the perpendicular linear distance from Cum to Plane CP.
6	BH	Height of CV body, the perpendicular linear distance from Cum to Plane CL.

**Table 2:** Desciption of the outcomes used in the objective staging system

No.	Outcome (Abbreviation)	Description
1	Superior Wall Inclination Angle (SWIA)	Angle between superior and posterior borders of C3 and C4 (CS^CP).
2	Concavity depth (CD)	Linear perpendicular distance from point Cd to plane CL.
3	Body Shape (BS)	Ratio of BW to BH of C3 and C4.

### Primary and secondary outcomes

**Fig. 3:**
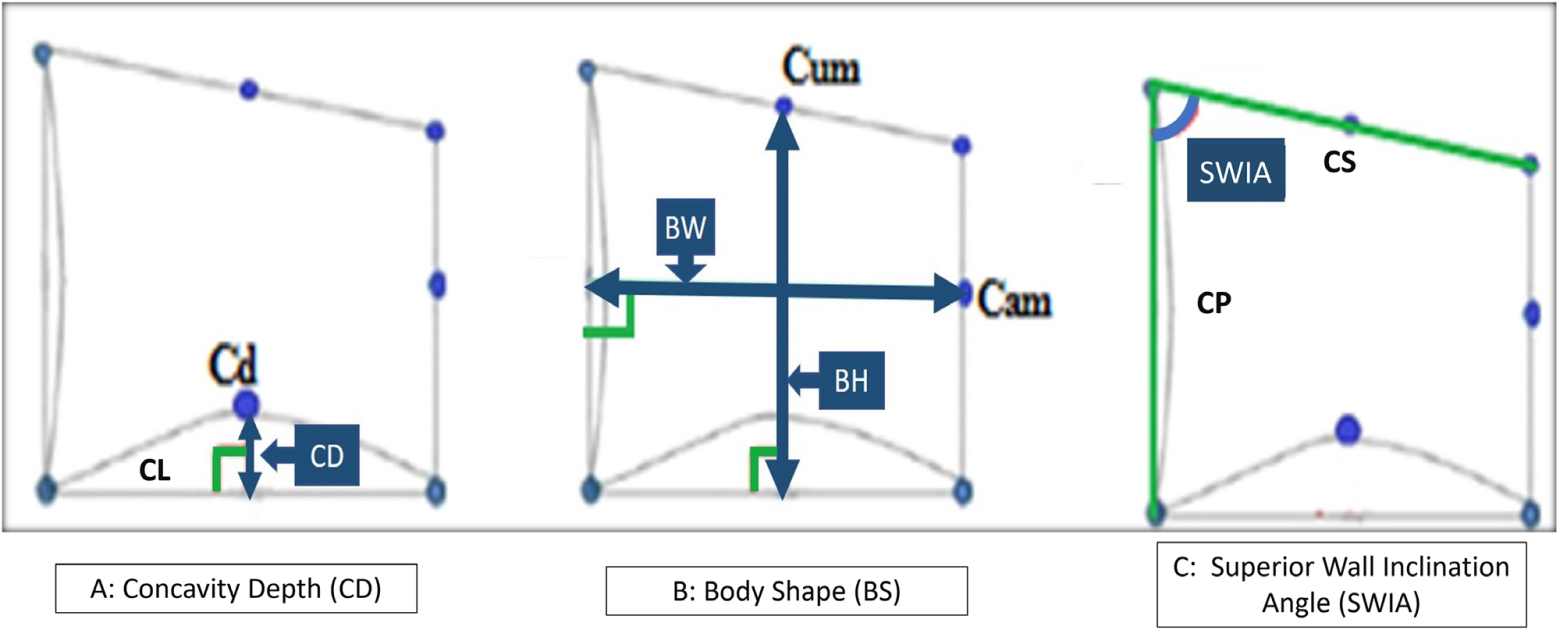
Objective staging system outcomes: (**A**) concavity depth (CD), the linear perpendicular distance from point Cd (most superior point of the inferior border of the body) to CL (the lower border) at C2, C3 and C4; (**B**) Body Shape, the ratio of Body width (BW) to Body height (BH) of C3 and C4; (**C**) Superior Wall Inclination Angle (SWIA), the angle between superior border (CS) and posterior border (CP) of C3 and C4

The primary outcome was the Superior Wall Inclination Angle (SWIA). The angle between the superior and posterior borders of C3 and C4. Secondary outcomes included Concavity Depth (CD) and Body Shape (BS). CD was the linear measurement from the deepest point at the lower borders C2, C3 and C4 to the line connecting the two inferior borders of C2,C3 and C4, respectively. BS was the ratio of body width (BW) to body height (BH) of C3 and C4 (Table 2; Fig. 3). Intra- and inter-observer reliability of the measurements were evaluated. Measurements for 10% of the sample were performed twice, 2 weeks apart, by the primary researcher (A.A) and an orthodontist (R.I).

### Statistical analysis

Statistical analysis was performed using IBM SPSS Statistics for Windows, Version 23 (Armonk, NY: IBM Corp). Quantitative data was explored for normality using Kolmogorov-Smirnov and Shapiro-Wilk tests. All data showed normal (parametric) distribution except for CD which showed non-normal (non-parametric) distribution. Non-parametric data was presented as median and range values, whereas parametric data was presented as mean and standard deviation (SD) values. For parametric data; a one-way ANOVA test followed by Bonferroni’s post-hoc test was used to compare between values of the outcomes at different stages. Non-parametric data was analyzed using Kruskal-Wallis test followed by Dunn’s test to make the comparisons between the outcomes at different stages. Qualitative data was presented as frequencies and percentages. Chi-square tests were used for the comparisons of qualitative variables. Intra and inter-observer reliability of the measurements were evaluated by Intraclass Correlation Coefficient (ICC). The significance level was set at P ≤ 0.05.

## Results

The initial sample included 647 LCRs. Matching the subjective evaluation scores decreased the sample to 394 LCRs. As seen in Fig. 1, 59, 220, and 115 LCRs were included in the pre-pubertal, pubertal, and post-pubertal stages, respectively.

According to the ICC analysis, intra- and inter-examiner reliability for subjective staging training sessions displayed high agreement between the four examiners. A reproducibility of 0.948 (95% confidence interval (CI), 0.900-0.973) for intra-observer reliability and 0.967 (95% CI, 0.945–0.982) for inter-observer reliability was revealed. Testing the reliability of linear and angular measurement revealed good agreement. Intra-observer reliability scored 0.918, 0.885 and 0.722 for CD, BS and SWIA respectively (95% CI). Inter-observer reliability results showed good agreement with scores of 0.902, 0.889 and 0.728 for CD, BS and SWIA respectively (95% CI). The findings are illustrated in Table 3.

**Table 3:** Intra and inter-observer reliability of the subjective staging system conducted by a panel of four examiners and the outcomes measured

Variables	Intra- observer reliability	Inter- observer reliability
Subjective assessment of the CVM	0.948	0.967
Reliability of the outcomes measured		
Outcome	Intra- observer reliability test	Inter- observer reliability test
Concavity Depth	0.918	0.902
Body shape	0.885	0.889
Superior Wall Inclination Angle	0.722	0.728

Mean and SD values of SWIA and BS at the pre-pubertal, pubertal, and post-pubertal stages were computed and compared. CD scores were computed in terms of Median and Range. The results are presented in Table 4.

**Table 4:** Descriptive statistics, results of one-way ANOVA and Kruskal-Wallis tests for comparison between outcome measurements of the pre-pubertal, pubertal and post-pubertal stages

Outcome	Vertebra	Pre-pubertal (n = 59)	Pubertal (n = 220)	Post-pubertal (n = 115)	*P*-value	*Effect size (Eta Squared)*
Superior Wall Inclination Angle [Mean (SD)] measured in degrees	C3	73.63 (3.72) ^C^	79.19 (4.23) ^B^	82.58 (3.84) ^A^	< 0.001	0.329
	C4	74.29 (3.98) ^C^	78.8 (3.77) ^B^	82.14 (4.18) ^A^	< 0.001	0.289
Concavity depth [Median (Range)] measured in mm	CD2	1 (0–1) ^B^	1.5 (0.5–2.5) ^AB^	2 (1–3) ^A^	< 0.001	0.402
	CD3	0 (0–0) ^B^	1.5 (0–3) ^A^	2 (1-3.5) ^A^	< 0.001	0.505
	CD4	0 (0–0) ^C^	1 (0-2.5) ^B^	2 (1–3) ^A^	< 0.001	0.464
Body width [Mean (SD)] measured in mm	C3	12.39 (1.16) ^B^	13.49 (1.34) ^A^	12.65 (1.1) ^B^	< 0.001	0.123
	C4	12.42 (1.13) ^C^	13.53 (1.35) ^A^	12.9 (1.13) ^B^	< 0.001	0.103
Body height [Mean (SD)] measured in mm	C3	7.47 (1.14) ^C^	10.19 (1.54) ^B^	12.86 (1) ^A^	< 0.001	0.627
	C4	7.47 (1.11) ^C^	9.95 (1.48) ^B^	12.42 (1.16) ^A^	< 0.001	0.59
Body Shape Ratio of BW to BH [Mean (SD)] measured as percentage	C3	1.69 (0.25) ^A^	1.35 (0.22) ^B^	0.99 (0.09) ^C^	< 0.001	0.584
	C4	1.69 (0.27) ^A^	1.38 (0.21) ^B^	1.04 (0.1) ^C^	< 0.001	0.538

The letters A, B and C indicated the differences in values between the stages, where A is the highest and C is the lowest value

1. SWIA: this angle was lowest at pre-pubertal stage (73.63° ±3.72 for C3 and 74.29°± 3.98 for C4). Higher values were recorded at the pubertal stage (79.19° ±4.23 for C3 and 78.8°±3.77 for C4). Compared to the previous stages, the post-pubertal stage values were significantly higher (82.58°±3.84 for C3 and 82.14°±4.18 for C4).
2. BS: significant differences were observed between the stages for BS of C3 (P-value < 0.001, Effect size = 0.584) and C4 (P-value < 0.001, Effect size = 0.538). Pair-wise comparisons between the stages revealed that the ratio significantly decreased from the pre-pubertal stage to the pubertal stage. The lowest value was recorded at the post-pubertal stage.
3. Concavity Depth (CD): Comparing CD values of the three pubertal stages revealed significant differences for C2 (P-value < 0.001, Effect size = 0.402), C3 (P-value < 0.001, Effect size = 0.505) and C4 (P-value < 0.001, Effect size = 0.464). According to pair-wise comparisons CD values of C2, C3, and C4 progressively increased from the pre-pubertal to the pubertal and post-pubertal stages.

## Discussion

The current study presented and evaluated a simple staging system. The null hypothesis was rejected. Linear and angular measurements objectively described and differentiated CVM stages. Cervical vertebral morphological features were applied to objectively distinguish the stages of maturation and estimate skeletal age of growing orthodontic patients. Individual maturational status could be determined using set values specific to pre-pubertal, pubertal and post-pubertal patients. The method helped resolve the issues related to reproducibility in the currently applied subjective CVM methods. McNamara and Franchi, stated that the the diminished reliability was caused by the absence of definitive description of the CVM stages [29]. Previously proposed objective methods presented complex formula that limited their clinical usefulness [24].

In the current study, the six stages of CVM were reduced to three stages; pre-pubertal (stages 1 and 2), pubertal (stages 3 and 4) and post-pubertal stages (stage 5 and 6). According to Baccetti et al. [9] the PGS occurs 2 years after CS1 and 1 year after CS2. Stages CS3 and CS4 were described as coinciding with the ascending portion of the PGS. The post-pubertal stages reached a year (for CS5) and 2 years (for CS6) from the PGS. From a clinical perspective, skeletal maturation evaluation is required for the detection of the peak of PGS, and the coinciding PHV of mandibular growth [2]. In simpler terms, it is required to state that the patient is pre-pubertal, pubertal or post-pubertal in order to decide on the timing and/or prognosis of the intervention required.

The sample of the current study was pooled according to the maturation status. Gender was not considered which may be highlighted as a major limiting factor to the validation and application of the proposed staging system. However, the methods of Baccetti et al. [9] and Hassel and Farman [7] included male and female participants and described the stages of maturation regardless of gender. Comparatively, objective staging studies presented separate equations for males and females [19, 27]. Another study [19] stated that gender influenced maturation stages [30]. Accordingly, the validation of the proposed staging system require separate evaluations for males and females.

Attention was paid to the reliability of staging throughout the study. The evaluation team achieved high reliability scores after the initial training and calibration. However, there was a marked attrition in the sample (394 from 647 cases). Such attrition would agree with studies that stated that subjective staging results in disagreement between observers [14, 15]. The images were randomly arranged for the evaluation team assessment to decrease expectation bias. Cropping the images to the area of the CV improved the assessment and eliminated the chance for correlating growth status of the patient from the CV with other visible indicators of maturational status such as dental age.

The outcomes evaluated were SWIA (at C3 and C4), BS (at C3 and C4) and CD (at C2, C3 and C4). The SWIA of C3 and C4 increased significantly from early to late stages. It is simple to apply and consistent with cephalometric measurements. Furthermore, it is an angular measurement and hence might not be affected by the size difference between genders and different communities. This angular measurement could also be a useful tool for artificial intelligence applications, automatic landmark identification and may enable automatic maturation evaluation more applicable.

Inferior border changes originally described by Baccetti et al. [9] as flat, concavity present and finally evident. Hassel and Farman [7] used the terms flat, developing, distinct, accentuated and finally deep. The description from both methods was clearly demonstrated in the current study and in numerical terms. The values of the measurements were statistically different when compared within each stage (values of C2, C3 and C4) and between stages. Accordingly, the average values may be an alternative to the terms in the original methods.

Cervical vertebral BS was originally described by Baccetti et al. [9] as trapezoidal, horizontally rectangular, squared and vertically rectangular. Hassel and Farman [7] referred to the change in shape with the consecutive terms tapered superior vertebral borders, more rectangular, rectangular, nearly square and finally square. In the current study BS, the ratio decreased, and the figures were significantly differentiated between the maturational stages. Baccetti et al. [9] compared cephalometric measurements of vertebral morphological characteristics in transition through maturation stages, the ratio between length of the base and anterior height of the vertebral bodies decreased. The current study presented numerical values that gave objectivity to the original descriptions. It may be stated that the simplicity of the current method would increase the reliability of assessment of maturation of orthodontic patients and thus makes the integration of maturational status in diagnosis more accurate. This would better identify and segregate pre-pubertal, pubertal and post-pubertal individuals. Diagnosis of patients in need for functional appliances therapy requiring targeted treatment in the pubertal stage (the period of active mandibular growth) would be identified with an objective method rather than a subjective method with reliability issues.

The limitations of the current study include the absence of a comparable skeletal maturation method, the retrospective nature of the sample, and the pooling of data with no reference to gender. Future studies that counteract these limitations are recommended. 

## Conclusions

An objective staging system was presented and evaluated. Individual maturational status could be determined using set values specific to pre-pubertal, pubertal and post-pubertal patients. Three outcomes (Superior wall inclination angle, concavity depth, and body shape) were unique objective identifiers of maturation. Due to the limitations of the current investigation, the proposed staging system and its set values should be considered for clinical application with caution. Further research is required to reduce the limitations evaluate the applicability, and gender differences.

## Data Availability

Data supporting the results reported are available with the principal investigator and the corresponding author. Please contact the corresponding author for such information that can be made available upon request.

## Abbreviations

CVM: Cervical vertebral maturation
PHV: Peak height velocity
PGS: Pubertal growth spurt
LCRs: Lateral cephalometric radiographs
CV: Cervical vertebrae
CBCT: Cone beam computed tomography
SWIA: Superior wall inclination angle
CD: Concavity depth
BS: Body shape
BW: Body width
BH: Body height
SD: Standard deviation
